# Expression quantitative trait loci for *PAX8* contributes to the prognosis of hepatocellular carcinoma

**DOI:** 10.1371/journal.pone.0173700

**Published:** 2017-03-24

**Authors:** Shijie Ma, Jianshui Yang, Ci Song, Zijun Ge, Jing Zhou, Guoxin Zhang, Zhibin Hu

**Affiliations:** 1 Department of Gastroenterology, Huai'an First People's Hospital, Nanjing Medical University, Nanjing, China; 2 Department of Epidemiology and Biostatistics, School of Public Health, Nanjing Medical University, Nanjing, China; 3 Jiangsu Key Lab of Cancer Biomarkers, Prevention and Treatment, Collaborative Innovation Center for Cancer Medicine, Nanjing Medical University, Nanjing,China; 4 Departmentof Gastroenterology, First Affiliated Hospital of Nanjing Medical University, Nanjing, China; Università degli Studi di Palermo, ITALY

## Abstract

Paired-box family member *PAX8* encodes a transcription factor that has a role in cell differentiation and cell growth and may participate in the prognosis of hepatocellular carcinoma (HCC). By bioinformatics analysis, we identified several single nucleotide polymorphisms (SNPs) within a newly identified long non-coding RNA (lncRNA) AC016683.6 as expression quantitative trait loci (eQTLs) for *PAX8*. Hence, we hypothesized that *PAX8*eQTLs in lncRNA AC016683.6 may influence the HCC prognosis. We then performed a case-only study to assess the association between the two SNPs as well as the prognosis of HCC in 331 HBV-positive HCC patients without surgical treatment. Cox proportional hazard models were used for survival analysis with adjustments for the age, gender, smoking status, drinking status, Barcelona-Clinic Liver Cancer (BCLC) stage, and chemotherapy or TACE (transcatheter hepatic arterial chemoembolization) status. We found that the G allele of rs1110839 and the T allele of rs4848320 in *PAX8*was significantly associated with a better prognosis compared with the T allele of rs1110839 and the C allele of rs4848320 (adjusted HR = 0.74, 95% CI = 0.61–0.91, *P* = 0.004 for rs1110839 and adjusted HR = 0.71, 95% CI = 0.54–0.94, *P* = 0.015 for rs4848320 in the additive model). Furthermore, the combined effect of the variant genotypes for these two SNPs was more prominent in patients with the BCLC-C stage orpatients with chemotherapy or TACE. Although the exact biological function remains to be explored, our findings suggest a possible association of *PAX8*eQTLs in lncRNA AC016683.6 with the HCC prognosis inthe Chinese population. Further large and functional studies are needed to confirm our findings.

## Introduction

Liver cancer is the fifth most common cancer worldwide and the third greatest cause of cancer-related death worldwide [1]. Hepatocellular carcinoma (HCC) is the most common type of liver cancer [2]. Although surgical resection, liver transplantation, radiotherapy and some other therapies are potentially effective treatments for HCC, studies have shown that HCC has an increasing incidence and a poor 5-year survival rate of approximately 7% despite treatment [3–5]. The Barcelona Clinic Liver Cancer (BCLC) staging classification has been widely endorsed to predict the prognosis of HCC patients; however, remarkably different survival outcomes among HCC patients at the same stage suggest that the existence of other important factors might affect the prognosis. This current status has inspired researchers to focus on identifying molecular biomarkers to guide individualized treatment and improve the prognosis of cancer patients.

Paired-box gene 8 (*PAX8*), a member of the PAX gene family, encodes a transcription factor that has a role in cell differentiation and cell growth, and silencing of *PAX8* in cell culture results in cell death [6]. *PAX8* is known to activate the transcription of *BCl2*, which is an anti-apoptotic gene that is also involved in *p53*suppression, suggesting it plays a role in tumor initiation and progression [7]. A recent study also showed that overexpression of *PAX8* protein by endometrial cancer is associated with poor disease outcomes[6]. Accumulating data have revealed that *PAX8*is expressed in a high percentage of kidney, thyroid, ovarian, and lung carcinomas [8–11]. However,the potential role of *PAX8* in HCC prognosis has been rarely explored.

Long non-coding RNAs (lncRNAs) are defined as non-protein-coding transcripts,which are usuallylonger than 200 nucleotides.Based on the recent studiesonlncRNAs, it is reasonable to believe thatlncRNAsare important for regulating gene expression in the nucleus, exerting their biological functions. Recent studieshave provideda comprehensive generalization on the functions of lncRNAsthatmay modulate transcription or post-transcription via targeting the splicing, stability, or translation of mRNAs [12]. LncRNAAC016683.6is located in the intron region of *PAX8*, which belongs to chromosome 2q13. Using bioinformatics analyses, we identified two single nucleotide polymorphisms (SNPs) (rs1110839 and rs4848320) in AC016683.6that may be the expression quantitative trait loci (eQTLs) for *PAX8* (http://www.regulomedb.org) [13]. Therefore, it is likely that the two SNPs could influence the interaction between AC016683.6 and *PAX8*, regulating the expression of *PAX8*. According to the potential role of *PAX8*, we hypothesized that *PAX8*eQTLsmayinfluence the development and progression of HCC. To validate our hypothesis, we examined the associations between the two SNPs of *PAX8*and the prognosis of 331patients with intermediate or advanced HCC in China.

## Materials and methods

### Study subjects

This study was approved by the institutional review board of Nanjing Medical University. All participants provided written informed consent to participate in this study and the ethics committees approved of this consent procedure.Theenrollment of participants was previously described[14,15]. In consideration for constructing a relatively homogenous population, our current study was restrained to HCC patients who did not undergo surgery in the intermediate stage (B) or advanced stage (C) according to the Barcelona Clinic Liver Cancer (BCLC) staging system [16,17]. Briefly, 414 intermediate or advanced HCC patients were consecutively recruited from Nantong Tumor Hospital and the First Affiliated Hospital of Nanjing Medical University, Jiangsu, China.All participants were newly diagnosed and histopathologically confirmed HCC cases. Then, we prospectively conducted a follow-up study every 3 months from the time of enrollment by personal or family contacts until death or final follow-up. As a result, 331 intermediate or advanced HCC patients who had completed follow-ups and whose clinical information was available were enrolled in our study with the response rate of 80.0%. The maximum follow-up time (MFT) for the 331 patients was 60.7 months (final follow-up in January 2013), and the median survival time (MST) was 14.5 months.

### Serological testing

After performingthe enzyme-linked immunosorbent assay(Kehua Bio-engineering Co., Ltd., Shanghai, China) according tothe manufacturer’s protocol[14], we detected HBsAg, anti-HBs, anti-HBc and anti-HCV in the serum collected from eachpatient respectively.

### Genotyping

Followingthe traditional method[18], genomic DNA was extracted from a leukocyte pellet by a series of treatments. Then, all SNPs were genotyped using the Sequenom Mass ARRAY iPLEX platform (SequenomInc). To reduce the false positive and error rates, three blank (water) controls were detected in each384-well plate during the sample testingevery time, and more than 10% samples were randomly repeated for quality control, checking whether the latter results coincided with the former. The success rates of genotyping for the two SNPs were all above 98%.

### Statistical analysis

The survival time was calculated from the date of HCC diagnosis to the date of patient death or the last follow-up. The associations between the median survival time(MST) and demographic characteristics, clinical features and genotypes were estimated using the Kaplan–Meier method and log-rank test. Univariate and multivariable Cox proportional hazard regression analyses were conducted to estimate the crude or adjusted hazard ratio (HR) and their 95% confidence intervals (CI) with adjustment for the age, gender, smoking status, drinking status, BCLC stage, and chemotherapy or TACE (transcatheter hepatic arterial chemoembolization) status. The Cox stepwise regression model was also performed to determine the predictive factors of HCC prognosis with a significance level of 0.050 for entering and 0.051 for removing the respective explanatory variables. The heterogeneity between subgroups was assessed with the chi-square-based Q-test, and the heterogeneity was considered significant for *P*<0.10.Analysiswas performed using Stata software (version 11.0;Stataconference, Chicago). All tests were two-sided, and the criterion of statistical significance was set at *P*<0.05.

## Results

### Patients’ characteristics and clinical features

The demographic characteristics and clinical information of the 331 HCC patients in stage B or C included in this study were previously described[15]. In brief, 258 of the 331 patients died from HCC, and 2 died from other causes during a period of up to 60.7 months of follow-up. For the disease-specific survival analysis, the latter were considered censored data in the analyses. Chemotherapy or TACE and the drinking status were significantly associated with the patient survival time (log-rank *P* ≤0.001 and 0.006 for the drinking status and chemotherapy or TACE status, respectively). Compared to those who received neither chemotherapy nor TACE therapy (MST = 3.4 months), patients with chemotherapy or TACE therapy (MST = 16.8 months) had a significantly decreased risk of death (61%, HR = 0.39; 95% CI = 0.29–0.51).

### Effects of *PAX8* polymorphisms on HCC survival

The associations of the two SNPs with HCC survival were examined in an additive model by the Kaplan–Meier method. As shown in Table 1, patients carrying rs1110839 GT/GG genotypes and rs4848320 CT/TT genotypes had a longer survival time(MST:14.3 months for rs1110839 GT/GG and 15.4 months for rs4848320 CT/TT) than those carrying the rs1110839 TT and rs4848320 CC genotypes (MST:13.4months for rs1110839 TT and 13.0 months for rs4848320 CC). Furthermore, multivariable Cox regression analysis showed that rs1110839 and rs4848320 could be considered significant prognostic markers for HCC (Table 1). After adjusting for the age, gender, smoking status, drinking status, BCLC stage, and chemotherapy or TACE status, variant genotypes of rs1110839 and rs4848320 were significantly associated with a favorable HCC prognosis (adjusted HR = 0.74, 95% CI = 0.61–0.91, *P* = 0.004 for rs1110839 andadjusted HR = 0.71, 95% CI = 0.54–0.94, *P* = 0.015 for rs4848320).

**Table 1:** Genotypes of two SNPs and HCC patients’ survival.

Genotype	PatientsN(%)	DeathsN(%)	MST(Mo)	CrudeHR(95%)	Adjusted HR^a^(95%CI)	*P*^a^
rs1110839						
TT	143(44.3)	123(48.4)	13.4	1	1	
GT	150(46.4)	109(42.9)	13.5	0.76(0.58–0.98)	0.68(0.52–0.89)	0.004
GG	30(9.3)	22(8.7)	16.0	0.71(0.45–1.12)	0.61(0.39–0.98)	0.039
GT/GG	180(55.7)	131(51.6)	14.3	0.75(0.58–0.96)	0.67(0.52–0.86)	0.002
Additive model				0.80(0.66–0.98)	0.74(0.61–0.91)	0.004
rs4848320						
CC	228(71.7)	183(72.9)	13.0	1	1	
CT	86(27.0)	65(25.9)	15.4	0.76(0.57–1.01)	0.67(0.50–0.89)	0.006
TT	4(1.3)	3(1.2)	9.5	1.10(0.35–3.44)	1.28(0.40–4.04)	0.679
CT/TT	90(28.3)	68(27.1)	15.4	0.77(0.58–1.01)	0.68(0.51–0.91)	0.008
Additive model				0.79(0.61–1.03)	0.71(0.54–0.94)	0.015

HCC: hepatocellular carcinoma; MST: median survival time; HR: hazard ratio; CI: confidence intervals.

^*a*^Adjusted for age, gender, smoking, drink, Chemotherapy/TACE and bclc stage.

### Stratified analysis and interaction effects

We then examined the combined effect of these two variants on the HCC survival and observed a significant locus-dose effect between favorable genotypes and the risk of death (*P* for trend <0.001). Compared to patients without favorable genotypes (MST = 12.6 months), those patients with 1 or 2–4 favorable genotypes had a significantly longer MST (13.3 and 14.9 months, respectively). After adjusting for the age, gender, smoking status, drinking status, BCLC stage, and chemotherapy or TACE status, patients with 1 or 2–4 favorable genotypes had 0.22- and 0.43-fold decreased risks of HCC-specific deaths, respectively (Table 2).

**Table 2:** Combined effect of two SNPs genotypes associated with HCC patients’ survival.

Combined genotypes(favorable genotypes carried)	Patientsn = 315	Deathsn = 249	MST(Months)	Adjusted HR^*a*^ (95% CI)	*P*^*a*^
0	135(42.9)	116(46.6)	12.6	1	
1	87(27.6)	65(26.1)	13.3	0.78 (0.57–1.07)	0.124
2–4	93(29.5)	68(27.3)	14.9	0.57 (0.42–0.77)	<0.001
trend				0.76 (0.66–0.89)	<0.001

HCC: hepatocellular carcinoma; MST: median survival time; HR: hazard ratio; CI: confidence intervals.rs1110839 “G” and rs4848320 “T” genotypes were considered as favorable loci.

^*a*^Adjusted for age, gender,smoking, drink,Chemotherapy/TACE and bclc stage.

Theassociations between *PAX8*SNPs and HCC survival were further investigated by stratified analysis of the age, gender, smoking status, drinking status, BCLC stage, and chemotherapy or TACE status. As shown in Table 3, we found that the protective effect of combined variant genotypes was more prominent in B stage patients and patients without chemotherapy and TACE (adjusted HR = 0.73, 95% CI = 0.62–0.86; adjusted HR = 0.66, 95% CI = 0.49–0.87, respectively) than in C stage patients or patients with chemotherapy or TACE therapy (adjusted HR = 1.30, 95% CI = 0.77–2.21 and adjusted HR = 0.96, 95% CI = 0.80–1.15; *P* = 0.040 and 0.026 for the heterogeneity test, respectively). Therefore, the gene-BCLC stage and gene-chemotherapy or TACE status interaction analysis were performed, and statistically significant multiplicative interactionswere observed, as shown in Tables 4 and 5 (*P* for multiplicative interaction = 0.029 and <0.001, respectively).

**Table 3:** Stratified analyses of combined effect of two SNPs genotypes associated with HCC patients’ survival.

Variables	Combined effect (Deaths/Patients)				*P*forheterogeneity
	0favorable genotype	1favorable genotype	2-4favorablegenotype	AdjustedHR^*a*^ (95% CI)	
Age					
≤53	67/76	28/37	39/52	0.89(0.73–1.08)	0.289
>53	49/59	37/50	29/41	0.75(0.60–0.95)	
Gender					
Male	97/114	58/78	57/78	0.79(0.67–0.93)	0.665
Female	19/21	7/9	11/15	0.87(0.58–1.32)	
Smoke					
No	46/53	20/29	23/35	0.72(0.56–0.94)	0.343
Yes	70/82	45/58	45/58	0.84(0.70–1.02)	
Drink					
No	47/55	18/30	29/40	0.79(0.63–1.01)	0.961
Yes	69/80	47/57	39/53	0.79(0.65–0.96)	
BCLC stage					
stage B	107/124	62/83	59/82	0.73(0.62–0.86)	0.040
stage C	9/11	3/4	9/11	1.30(0.77–2.21)	
Chemotherapy/TACE					
No	44/46	15/20	17/23	0.66(0.49–0.87)	0.026
Yes	72/89	50/67	51/70	0.96(0.80–1.15)	

HR: hazard ratio; CI: confidence intervals.

^*a*^Adjusted for age, gender,smoking, drink,Chemotherapy/TACE and bclc stage.

**Table 4:** Interactive effect of combined effect of two SNPs genotypes and bclc stage associated with HCC patients’ survival.

BCLCstage	No. of favorable genotype	Patients	Deaths	Adjusted HR^*a*^(95%CI)	*P*^*a*^
		N (%)	N (%)		
3	0	11(3.5)	9(3.6)	1	
3	1	4(1.3)	3(1.2)	1.35(0.36–5.11)	0.658
3	2–4	11(3.5)	9(3.6)	1.52(0.60–3.86)	0.378
2	0	124(39.4)	107(43.0)	1.74(0.87–3.47)	0.116
2	1	83(26.3)	62(24.9)	1.34(0.66–2.72)	0.425
2	2–4	82(26.0)	59(23.7)	0.91(0.44–1.85)	0.789
trend				0.90(0.82–0.99)	0.029

HCC: hepatocellular carcinoma;HR: hazard ratio; CI: confidence intervals.

^*a*^Adjusted for age, gender,smoking, drink,Chemotherapy/TACE.

**Table 5:** Interactive effect of combined effect of two SNPs genotypes and Chemotherapy/TACE associated with HCC patients’ survival.

Chemotherapy/TACE	No. of favorable genotype	Patients	Deaths	Adjusted HR^*a*^(95%CI)	*P*^*a*^
		N (%)	N (%)		
No	0	46(14.6)	44(17.7)	1	
No	1	20(6.3)	15(6.0)	0.36(0.19–0.65)	0.001
No	2–4	23(7.3)	17(6.8)	0.22(0.13–0.40)	<0.001
Yes	0	89(28.3)	72(28.9)	0.15(0.10–0.23)	<0.001
Yes	1	67(21.3)	50(20.1)	0.17(0.11–0.26)	<0.001
Yes	2–4	70(22.2)	51(20.5)	0.13(0.09–0.21)	<0.001
trend				0.69(0.63–0.76)	<0.001

HCC: hepatocellular carcinoma;HR: hazard ratio; CI: confidence intervals.

^*a*^Adjusted for age, gender,smoking, drink, and bclc stage.

### Stepwise Cox regression analyses

We finally performed stepwise Cox proportional hazard analysis to evaluate the effects of the age, gender, smoking status, drinking status, BCLC stage, and chemotherapy or TACE status and *PAX8*eQTLs on HCC survival. Four variables (Chemotherapy or TACE status, age, drinking status and *PAX8*eQTLs) were selectedfor use in the final regression model. Furthermore, when the gender, drinking status, chemotherapy or TACE status, and *PAX8*eQTLswere included in the final model, the *PAX8*eQTLs remained an independent protective factor for HCC survival (HR = 0.78, 95% CI = 0.67–0.91, *P* = 0.001)(Table 6).

**Table 6:** Multivariate Cox regression analysis on HCC patients’ survival.

Variables	β^*a*^	SE^*b*^	HR (95% CI)	*P*
Stepwise regression analysis				
Chemotherapy or TACE (yes vs. no)	-1.1804	0.0464	0.31 (0.23–0.41)	<0.001
Combined genotypes (1 or 2–4 favorable genotypes vs 0 favorable genotypes)	-0.2471	0.0598	0.78 (0.67–0.91)	0.001
Age (>53 vs. < = 53)	-0.4208	0.0892	0.66 (0.50–0.86)	0.002
Drinking status (yes vs. no)	0.4004	0.1969	1.49 (1.15–1.93)	0.002
Final regression model				
Combined genotypes (1 or 2–4 favorable genotypes vs 0 favorable genotypes)	-0.2471	0.0598	0.78 (0.67–0.91)	0.001
Age (>53 vs. < = 53)	-0.4208	0.0892	0.66 (0.50–0.86)	0.002
Drinking status (yes vs. no)	0.4004	0.1969	1.49(1.15–1.93)	0.002
Chemotherapy or TACE (yes vs. no)	-1.1804	0.0464	0.31 (0.23–0.41)	<0.001

^a^ β is the estimated parameter of the regression model.

^b^ SE is the standard error of the regression model.

## Discussion

In the present case-cohort study, we demonstrated that *PAX8*eQTLs (rs1110839 and rs4848320) may be an independent candidate biomarker for predicting HCC survival in Chinese with a prospective study design. Furthermore, the combined effect of these two SNPs was more prominent in patients with BCLC-B stage who did not receivechemotherapy or TACE.

Since the ENCODE Project and RNA-seq analysis have identified thousands of new lncRNAs, the genetic variants and biological function of lncRNAs are becoming a focus in studies of complex diseases. We previously reported that *ZNRD1*(Zinc ribbon domain containing 1)eQTLsincreased the risk of HCCLncRNA AC016683.6, located in the intron region of *PAX8*, may influence the expression of *PAX8*. A PAX family member, *PAX8*, is implicated in the development of the kidney, thyroid gland, Mullerian and Wolffian ducts, and others [19,20]. Tumors derived from these organs, as mentioned above, also typically express PAX8[21]. One study showed that PAX8 activates the transcription of the *NCAM* gene through binding sequences resembling paired domain binding sites in the *NCAM* promoter [22]. *NCAM*is expressed on the surface of immune cells [23], and it has been shown to mediate adhesion between immune cells, which is particularly important in inflammation [24]. Previous studies have shown that abnormal cell growth and proliferation are often associated with high expression levels of *PAX* genes. In fact, overexpression of PAX proteins does not appear to be an initiating or transforming molecular event in tumor pathogenesis, but it facilitates malignant development through the effects of *PAX* genes on apoptosis resistance, tumor cell proliferation and migration, and repression of terminal differentiation [25]. Our results showed that the variant genotypes (rs1110839 GT/GG and rs4848320 CT/TT genotypes) of the two *PAX8*eQTLs in lncRNAAC016683.6promotedbetter prognosis in HCC patients with intermediate or advanced disease.

There are several limitations ofthe study that need to be addressed by further research. First, further validation needs to be conducted. Large-scale studies are required to validate the associations between the two eQTLs in AC016683.6 and the HCC prognosis. Second, the biological function of the two eQTLsin AC016683.6 was not further investigated in this study. Additionally, our previous study found that *ZNRD1*eQTLs rs3757328 in *ZNRD1-AS1* (*ZNRD1* antisense RNA1) was associated with an increased risk for HCC, and further eQTL analysis indicated the significant association between the genotypes of rs3757328 and the expression of *ZNRD1* and *ZNRD1-AS1*. In vitro experiments have also demonstrated that *ZNRD1* knockdown inhibits the expression of HBV mRNA and promotes the proliferation of HepG2.2.15 cells. Given the findings from our previous study[26], we hypothesized that lncRNAAC016683.6 might regulate the expression of a related protein (PAX8) based on its variation, influencing the prognosis of hepatic tumors.

To the best of ourknowledge, this is the first study investigating the association between *PAX8*eQTLs in the lncRNAAC016683.6andHCC prognosis. This study showed that the variant rs1110839 GT/GG and rs4848320 CT/TT genotypes in lncRNA AC016683.6 influenced the prognosis of hepatic tumor patients. These results suggested that AC016683.6 rs1110839 and rs4848320 might serve as susceptibility markers for HCC survival. Further studies incorporating diverse populations and functional assays are warranted to validate and extend our findings.

## Supporting information

S1 Data SNP genotying data of the study. (PDF)
