# Supplementary material for: Expression quantitative trait loci for PAX8 contributes to the prognosis of hepatocellular carcinoma
Source: PLoS One. 2017 Mar 24;12(3):e0173700. doi: 10.1371/journal.pone.0173700 (PMC5365105; doi:10.1371/journal.pone.0173700)
Supplement: S1 Data — (PDF) [file pone.0173700.s001.pdf]

Number	Type	sex	age	rs1110839	rs4848320
1	hepatocellular carcinoma	man	34	GG	CC
2	hepatocellular carcinoma	man	51	GG	TT
3	hepatocellular carcinoma	man	44	GG	CT
4	hepatocellular carcinoma	man	62	GG	CT
5	hepatocellular carcinoma	man	49	GG	CT
6	hepatocellular carcinoma	man	56	GG	CT
7	hepatocellular carcinoma	man	43	GG	CT
8	hepatocellular carcinoma	man	39	GG	
9	hepatocellular carcinoma	man	74	GG	TT
10	hepatocellular carcinoma	man	53	GG	CT
11	hepatocellular carcinoma	woman	37	GG	CC
12	hepatocellular carcinoma	woman	46	GG	CT
13	hepatocellular carcinoma	man	67	GG	TT
14	hepatocellular carcinoma	man	47	GG	TT
15	hepatocellular carcinoma	man	55	GG	CC
16	hepatocellular carcinoma	man	78	GG	CC
17	hepatocellular carcinoma	woman	53	GG	CC
18	hepatocellular carcinoma	woman	62	GG	CT
19	hepatocellular carcinoma	man	52	GG	CC
20	hepatocellular carcinoma	man	34	GG	CT
21	hepatocellular carcinoma	man	52	GG	CC
22	hepatocellular carcinoma	man	58	GG	CT
23	hepatocellular carcinoma	man	59	GG	CT
24	hepatocellular carcinoma	man	59	GG	CC
25	hepatocellular carcinoma	man	66	GG	CT
26	hepatocellular carcinoma	man	42	GG	CC
27	hepatocellular carcinoma	man	71	GG	CT
28	hepatocellular carcinoma	man	58	GG	CT
29	hepatocellular carcinoma	man	54	GG	CT
30	hepatocellular carcinoma	man	39	GG	CC
31	hepatocellular carcinoma	man	50	GT	
32	hepatocellular carcinoma	man	55	GT	
33	hepatocellular carcinoma	man	55	GT	
34	hepatocellular carcinoma	man	55	GT	CC
35	hepatocellular carcinoma	man	66	GT	CC
36	hepatocellular carcinoma	man	62	GT	CC
37	hepatocellular carcinoma	man	53	GT	CC
38	hepatocellular carcinoma	woman	47	GT	CC
39	hepatocellular carcinoma	man	41	GT	CT
40	hepatocellular carcinoma	man	44	GT	CT
41	hepatocellular carcinoma	man	65	GT	CT
42	hepatocellular carcinoma	man	65	GT	CT
43	hepatocellular carcinoma	man	47	GT	CT
44	hepatocellular carcinoma	man	67	GT	CC
45	hepatocellular carcinoma	man	49	GT	CT
46	hepatocellular carcinoma	man	46	GT	CT
47	hepatocellular carcinoma	man	40	GT	CT
48	hepatocellular carcinoma	man	60	GT	CC
49	hepatocellular carcinoma	man	58	GT	CC
50	hepatocellular carcinoma	man	64	GT	CC
51	hepatocellular carcinoma	man	37	GT	CC
52	hepatocellular carcinoma	woman	55	GT	CT
53	hepatocellular carcinoma	man	46	GT	
54	hepatocellular carcinoma	man	52	GT	CC

55	hepatocellular carcinoma	man	62	GT	CC
56	hepatocellular carcinoma	man	53	GT	CT
57	hepatocellular carcinoma	man	47	GT	CC
58	hepatocellular carcinoma	man	45	GT	CC
59	hepatocellular carcinoma	man	55	GT	CC
60	hepatocellular carcinoma	man	65	GT	CC
61	hepatocellular carcinoma	man	43	GT	CC
62	hepatocellular carcinoma	man	45	GT	CC
63	hepatocellular carcinoma	man	47	GT	CC
64	hepatocellular carcinoma	man	44	GT	CC
65	hepatocellular carcinoma	man	47	GT	CT
66	hepatocellular carcinoma	man	49	GT	CT
67	hepatocellular carcinoma	man	68	GT	CT
68	hepatocellular carcinoma	man	57	GT	CC
69	hepatocellular carcinoma	man	61	GT	CT
70	hepatocellular carcinoma	man	41	GT	CT
71	hepatocellular carcinoma	woman	61	GT	CC
72	hepatocellular carcinoma	woman	39	GT	CC
73	hepatocellular carcinoma	man	44	GT	CT
74	hepatocellular carcinoma	man	55	GT	CT
75	hepatocellular carcinoma	man	58	GT	CC
76	hepatocellular carcinoma	man	63	GT	CC
77	hepatocellular carcinoma	man	51	GT	CT
78	hepatocellular carcinoma	man	54	GT	CC
79	hepatocellular carcinoma	man	44	GT	CC
80	hepatocellular carcinoma	man	70	GT	CC
81	hepatocellular carcinoma	man	32	GT	CT
82	hepatocellular carcinoma	woman	58	GT	CT
83	hepatocellular carcinoma	man	62	GT	CC
84	hepatocellular carcinoma	man	58	GT	CT
85	hepatocellular carcinoma	man	47	GT	CT
86	hepatocellular carcinoma	man	69	GT	CC
87	hepatocellular carcinoma	man	68	GT	CC
88	hepatocellular carcinoma	man	50	GT	
89	hepatocellular carcinoma	man	50	GT	CC
90	hepatocellular carcinoma	man	66	GT	CC
91	hepatocellular carcinoma	man	47	GT	CT
92	hepatocellular carcinoma	man	45	GT	CC
93	hepatocellular carcinoma	man	37	GT	CC
94	hepatocellular carcinoma	man	45	GT	CC
95	hepatocellular carcinoma	man	25	GT	CT
96	hepatocellular carcinoma	woman	76	GT	CC
97	hepatocellular carcinoma	man	47	GT	CC
98	hepatocellular carcinoma	man	57	GT	CC
99	hepatocellular carcinoma	man	63	GT	CC
100	hepatocellular carcinoma	man	54	GT	CT
101	hepatocellular carcinoma	woman	35	GT	CT
102	hepatocellular carcinoma	man	73	GT	CT
103	hepatocellular carcinoma	man	39	GT	CC
104	hepatocellular carcinoma	man	43	GT	CT
105	hepatocellular carcinoma	man	55	GT	CT
106	hepatocellular carcinoma	man	56	GT	CC
107	hepatocellular carcinoma	woman	63	GT	CC
108	hepatocellular carcinoma	man	40	GT	CC
109	hepatocellular carcinoma	man	58	GT	CC

110	hepatocellular carcinoma	man	83	GT	CC
111	hepatocellular carcinoma	man	39	GT	CT
112	hepatocellular carcinoma	man	51	GT	CT
113	hepatocellular carcinoma	man	49	GT	CT
114	hepatocellular carcinoma	man	65	GT	CT
115	hepatocellular carcinoma	man	72	GT	CC
116	hepatocellular carcinoma	man	43	GT	CT
117	hepatocellular carcinoma	man	48	GT	CT
118	hepatocellular carcinoma	man	44	GT	CT
119	hepatocellular carcinoma	man	74	GT	CC
120	hepatocellular carcinoma	man	59	GT	CT
121	hepatocellular carcinoma	man	64	GT	CC
122	hepatocellular carcinoma	man	67	GT	CC
123	hepatocellular carcinoma	man	53	GT	CT
124	hepatocellular carcinoma	man	69	GT	CC
125	hepatocellular carcinoma	woman	43	GT	CT
126	hepatocellular carcinoma	man	42	GT	CT
127	hepatocellular carcinoma	man	50	GT	CC
128	hepatocellular carcinoma	man	40	GT	CC
129	hepatocellular carcinoma	woman	57	GT	CT
130	hepatocellular carcinoma	man	62	GT	CC
131	hepatocellular carcinoma	man	59	GT	CT
132	hepatocellular carcinoma	man	64	GT	CT
133	hepatocellular carcinoma	man	52	GT	CC
134	hepatocellular carcinoma	man	50	GT	CC
135	hepatocellular carcinoma	woman	37	GT	CT
136	hepatocellular carcinoma	woman	63	GT	CT
137	hepatocellular carcinoma	man	34	GT	CT
138	hepatocellular carcinoma	man	40	GT	CC
139	hepatocellular carcinoma	man	44	GT	CC
140	hepatocellular carcinoma	man	51	GT	CT
141	hepatocellular carcinoma	man	56	GT	CT
142	hepatocellular carcinoma	man	63	GT	CT
143	hepatocellular carcinoma	man	66	GT	CC
144	hepatocellular carcinoma	man	55	GT	CC
145	hepatocellular carcinoma	man	56	GT	CT
146	hepatocellular carcinoma	man	51	GT	CT
147	hepatocellular carcinoma	man	54	GT	CT
148	hepatocellular carcinoma	woman	59	GT	CC
149	hepatocellular carcinoma	woman	89	GT	CT
150	hepatocellular carcinoma	man	46	GT	CC
151	hepatocellular carcinoma	man	57	GT	CC
152	hepatocellular carcinoma	woman	50	GT	CT
153	hepatocellular carcinoma	man	61	GT	CC
154	hepatocellular carcinoma	man	65	GT	CC
155	hepatocellular carcinoma	man	50	GT	CT
156	hepatocellular carcinoma	man	27	GT	CC
157	hepatocellular carcinoma	man	74	GT	CC
158	hepatocellular carcinoma	man	37	GT	CC
159	hepatocellular carcinoma	woman	62	GT	CC
160	hepatocellular carcinoma	man	55	GT	CC
161	hepatocellular carcinoma	man	60	GT	CC
162	hepatocellular carcinoma	man	37	GT	CC
163	hepatocellular carcinoma	man	50	GT	CC
164	hepatocellular carcinoma	man	55	GT	CC

165	hepatocellular carcinoma	man	53	GT	CT
166	hepatocellular carcinoma	man	56	GT	CT
167	hepatocellular carcinoma	man	54	GT	CT
168	hepatocellular carcinoma	woman	50	GT	CT
169	hepatocellular carcinoma	man	54	GT	CC
170	hepatocellular carcinoma	woman	41	GT	CT
171	hepatocellular carcinoma	man	55	GT	CC
172	hepatocellular carcinoma	man	51	GT	CC
173	hepatocellular carcinoma	man	61	GT	CT
174	hepatocellular carcinoma	woman	53	GT	CC
175	hepatocellular carcinoma	man	63	GT	CT
176	hepatocellular carcinoma	man	38	GT	CT
177	hepatocellular carcinoma	man	55	GT	CC
178	hepatocellular carcinoma	man	64	GT	CC
179	hepatocellular carcinoma	woman	59	GT	CC
180	hepatocellular carcinoma	man	59	GT	CT
181	hepatocellular carcinoma	man	58	TT	
182	hepatocellular carcinoma	man	55	TT	
183	hepatocellular carcinoma	man	61	TT	CT
184	hepatocellular carcinoma	man	51	TT	CC
185	hepatocellular carcinoma	woman	53	TT	CC
186	hepatocellular carcinoma	man	37	TT	CC
187	hepatocellular carcinoma	man	46	TT	CC
188	hepatocellular carcinoma	man	56	TT	CC
189	hepatocellular carcinoma	man	71	TT	CC
190	hepatocellular carcinoma	man	48	TT	CC
191	hepatocellular carcinoma	man	33	TT	CC
192	hepatocellular carcinoma	man	47	TT	CC
193	hepatocellular carcinoma	man	56	TT	CC
194	hepatocellular carcinoma	man	29	TT	CC
195	hepatocellular carcinoma	man	46	TT	CC
196	hepatocellular carcinoma	woman	50	TT	CC
197	hepatocellular carcinoma	man	51	TT	CT
198	hepatocellular carcinoma	man	57	TT	CC
199	hepatocellular carcinoma	man	44	TT	CC
200	hepatocellular carcinoma	man	66	TT	CC
201	hepatocellular carcinoma	woman	47	TT	CC
202	hepatocellular carcinoma	man	53	TT	CC
203	hepatocellular carcinoma	man	71	TT	CC
204	hepatocellular carcinoma	man	46	TT	CC
205	hepatocellular carcinoma	man	38	TT	CC
206	hepatocellular carcinoma	man	49	TT	CC
207	hepatocellular carcinoma	woman	58	TT	CC
208	hepatocellular carcinoma	man	40	TT	CC
209	hepatocellular carcinoma	woman	68	TT	CC
210	hepatocellular carcinoma	man	42	TT	CC
211	hepatocellular carcinoma	man	71	TT	CC
212	hepatocellular carcinoma	man	53	TT	CC
213	hepatocellular carcinoma	man	49	TT	CC
214	hepatocellular carcinoma	man	58	TT	CC
215	hepatocellular carcinoma	man	46	TT	CC
216	hepatocellular carcinoma	woman	54	TT	CC
217	hepatocellular carcinoma	man	38	TT	CC
218	hepatocellular carcinoma	man	43	TT	CC
219	hepatocellular carcinoma	man	46	TT	CC

220	hepatocellular carcinoma	man	71	TT	CC
221	hepatocellular carcinoma	man	65	TT	CC
222	hepatocellular carcinoma	man	45	TT	CC
223	hepatocellular carcinoma	man	58	TT	CC
224	hepatocellular carcinoma	man	43	TT	CC
225	hepatocellular carcinoma	man	40	TT	CC
226	hepatocellular carcinoma	woman	52	TT	CC
227	hepatocellular carcinoma	woman	42	TT	CC
228	hepatocellular carcinoma	woman	43	TT	CC
229	hepatocellular carcinoma	woman	51	TT	CC
230	hepatocellular carcinoma	man	42	TT	CC
231	hepatocellular carcinoma	man	43	TT	CC
232	hepatocellular carcinoma	man	46	TT	CC
233	hepatocellular carcinoma	man	46	TT	CC
234	hepatocellular carcinoma	man	52	TT	CC
235	hepatocellular carcinoma	man	54	TT	CC
236	hepatocellular carcinoma	man	43	TT	CC
237	hepatocellular carcinoma	man	65	TT	CC
238	hepatocellular carcinoma	man	71	TT	CC
239	hepatocellular carcinoma	woman	55	TT	CC
240	hepatocellular carcinoma	man	68	TT	CC
241	hepatocellular carcinoma	man	47	TT	CC
242	hepatocellular carcinoma	man	51	TT	CC
243	hepatocellular carcinoma	man	78	TT	CC
244	hepatocellular carcinoma	man	54	TT	CC
245	hepatocellular carcinoma	man	62	TT	CC
246	hepatocellular carcinoma	man	34	TT	CC
247	hepatocellular carcinoma	man	61	TT	CC
248	hepatocellular carcinoma	man	62	TT	CC
249	hepatocellular carcinoma	man	61	TT	CC
250	hepatocellular carcinoma	man	61	TT	CC
251	hepatocellular carcinoma	man	44	TT	CC
252	hepatocellular carcinoma	woman	57	TT	CC
253	hepatocellular carcinoma	man	38	TT	CC
254	hepatocellular carcinoma	man	57	TT	CC
255	hepatocellular carcinoma	man	40	TT	CC
256	hepatocellular carcinoma	man	21	TT	CC
257	hepatocellular carcinoma	man	52	TT	CT
258	hepatocellular carcinoma	woman	53	TT	CC
259	hepatocellular carcinoma	man	37	TT	CC
260	hepatocellular carcinoma	man	54	TT	CC
261	hepatocellular carcinoma	man	48	TT	CC
262	hepatocellular carcinoma	man	73	TT	CC
263	hepatocellular carcinoma	man	44	TT	CC
264	hepatocellular carcinoma	man	49	TT	CC
265	hepatocellular carcinoma	man	45	TT	CT
266	hepatocellular carcinoma	man	51	TT	CC
267	hepatocellular carcinoma	man	69	TT	CC
268	hepatocellular carcinoma	woman	39	TT	CC
269	hepatocellular carcinoma	man	54	TT	CC
270	hepatocellular carcinoma	man	42	TT	CC
271	hepatocellular carcinoma	man	78	TT	CC
272	hepatocellular carcinoma	man	48	TT	CC
273	hepatocellular carcinoma	man	64	TT	CC
274	hepatocellular carcinoma	man	58	TT	CC

275	hepatocellular carcinoma	woman	42	TT	CC
276	hepatocellular carcinoma	man	55	TT	CC
277	hepatocellular carcinoma	man	71	TT	CC
278	hepatocellular carcinoma	woman	72	TT	CC
279	hepatocellular carcinoma	man	52	TT	CT
280	hepatocellular carcinoma	man	43	TT	CC
281	hepatocellular carcinoma	man	54	TT	CC
282	hepatocellular carcinoma	man	39	TT	CC
283	hepatocellular carcinoma	man	58	TT	CC
284	hepatocellular carcinoma	woman	73	TT	CC
285	hepatocellular carcinoma	man	50	TT	CT
286	hepatocellular carcinoma	man	53	TT	CC
287	hepatocellular carcinoma	man	38	TT	CC
288	hepatocellular carcinoma	man	52	TT	CC
289	hepatocellular carcinoma	man	70	TT	CC
290	hepatocellular carcinoma	man	53	TT	CC
291	hepatocellular carcinoma	man	61	TT	CC
292	hepatocellular carcinoma	woman	56	TT	CC
293	hepatocellular carcinoma	man	43	TT	CC
294	hepatocellular carcinoma	man	53	TT	CC
295	hepatocellular carcinoma	man	54	TT	CC
296	hepatocellular carcinoma	woman	53	TT	CC
297	hepatocellular carcinoma	man	43	TT	CC
298	hepatocellular carcinoma	man	70	TT	CC
299	hepatocellular carcinoma	man	38	TT	CC
300	hepatocellular carcinoma	man	57	TT	CC
301	hepatocellular carcinoma	man	42	TT	CC
302	hepatocellular carcinoma	man	49	TT	CC
303	hepatocellular carcinoma	man	49	TT	CC
304	hepatocellular carcinoma	man	69	TT	CC
305	hepatocellular carcinoma	man	49	TT	CC
306	hepatocellular carcinoma	man	61	TT	CC
307	hepatocellular carcinoma	man	63	TT	CC
308	hepatocellular carcinoma	man	66	TT	CC
309	hepatocellular carcinoma	man	68	TT	CC
310	hepatocellular carcinoma	woman	55	TT	CC
311	hepatocellular carcinoma	man	51	TT	CC
312	hepatocellular carcinoma	man	54	TT	CC
313	hepatocellular carcinoma	man	59	TT	CC
314	hepatocellular carcinoma	woman	64	TT	CC
315	hepatocellular carcinoma	man	53	TT	CC
316	hepatocellular carcinoma	man	63	TT	CC
317	hepatocellular carcinoma	man	46	TT	CC
318	hepatocellular carcinoma	man	67	TT	CC
319	hepatocellular carcinoma	man	38	TT	CC
320	hepatocellular carcinoma	man	53	TT	CC
321	hepatocellular carcinoma	man	61	TT	CC
322	hepatocellular carcinoma	man	38	TT	CC
323	hepatocellular carcinoma	man	52	TT	CC
324	hepatocellular carcinoma	man	47		
325	hepatocellular carcinoma	man	27		
326	hepatocellular carcinoma	woman	76		
327	hepatocellular carcinoma	woman	55		
328	hepatocellular carcinoma	man	52		
329	hepatocellular carcinoma	man	66		CC

330	hepatocellular carcinoma	man	68	CT
331	hepatocellular carcinoma	man	39	CC
